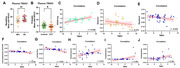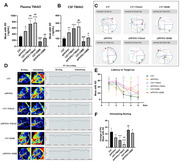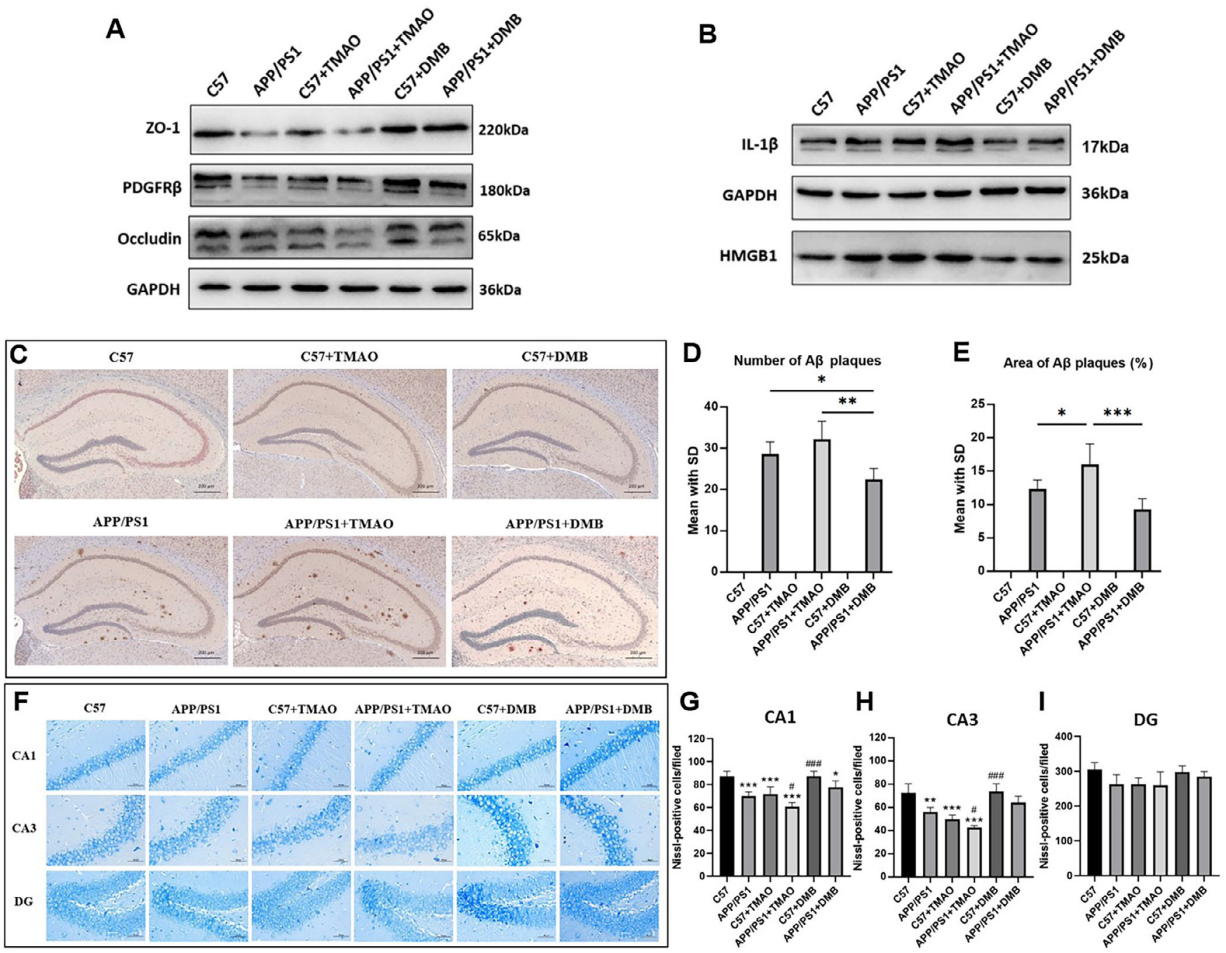# Trimethylamine‐N‐Oxide regulates cognitive function in Alzheimer’s Disease patients and mice model through brain‐gut‐microbiota axis

**DOI:** 10.1002/alz.086101

**Published:** 2025-01-09

**Authors:** Wenbo Zhang, Yang Lü

**Affiliations:** ^1^ The First Affiliated Hospital of Chongqing Medical University, Chongqing, Chongqing China

## Abstract

**Background:**

The regulatory role of Trimethylamine‐N‐oxide (TMAO) for cognition from the perspective of microbiota‐gut‐brain (MGB) axis in AD remains unclear.

**Method:**

In clinical cohort study for effects of 24‐week computerized cognitive training (CCT), registered on clinicaltrials.gov (NCT06094452), plasma TMAO levels were quantified using ELISA in MCI (n=39) and mild AD patients (n=35). Correlation analyses were conducted to assess the relationship between changes in TMAO levels and cognitive function (ADAS‐cog score) and gut microbiota indices. In animal model experiments, C57/BL6 aged mice and APP/PS1 transgenic mice were administrated by water feeding with 1.2% TMAO for 16 weeks and 1.0% of its inhibitor 3,3‐Dimethyl‐1‐butanol (DMB) for 8 weeks. TMAO levels in plasma and cerebrospinal fluid (CSF) were measured by ELISA. Cerebral blood flow (CBF) activation was detected by a laser speckle imaging system. Cognitive behavior was evaluated using Morris Water Maze test. The expressions of BBB function proteins (ZO‐1, Occludin, and PDGFRβ,) and neuroinflammatory markers (HMGB1 and IL‐1β) were detected by western blotting. The deposition of β‐amyloid (Aβ) plaques and neuronal survival in hippocampus were visualized by histochemical and Nissl staining.

**Result:**

TMAO was significantly increased in plasma of AD patients. After CCT intervention, TMAO was significantly decreased in the CCT group, and this decrease was positively correlated with ADAS‐cog (r=0.549), and negatively correlated with Shannon evenness (r= ‐0.372). TMAO changes were also negatively correlated with the relative abundance change of Roseburia (r=‐0.443), Lachnospiraceae_UCG‐004 (r=‐0.392), and Lactobacillus (r=0.480), while positively correlated with Ruminococcus_torgues (r=0.443), Fusobacterium (r=0.484) and Herbasporillum (r=0.365). In animal models, TMAO was increased in the plasma and CSF of APP/PS1 mice. After supplemented with 1.2% TMAO, the cognitive performances of C57 and APP/PS1 mice were significantly impaired. The activation amplitude of CBF was decreased, and the expression of BBB function proteins (ZO‐1, Occludin, and PDGFRβ) were significantly decreased. The expressions of neuroinflammation marker HMGB1 and IL‐1β were significantly increased; the accumulation of Aβplaques was increased with decreased neuronal survival. Circulating level of TMAO was decreased after DMB intervention, which showed cognitive protection effects.

**Conclusion:**

TMAO may be a novel biomarker that regulates cognition of AD through MGB axis.